# Genetic Characterization of Methicillin-Resistant *Staphylococcus aureus* Isolates from Human Bloodstream Infections: Detection of MLS_B_ Resistance

**DOI:** 10.3390/antibiotics9070375

**Published:** 2020-07-03

**Authors:** Vanessa Silva, Sara Hermenegildo, Catarina Ferreira, Célia M. Manaia, Rosa Capita, Carlos Alonso-Calleja, Isabel Carvalho, José Eduardo Pereira, Luis Maltez, José L. Capelo, Gilberto Igrejas, Patrícia Poeta

**Affiliations:** 1 Microbiology and Antibiotic Resistance Team (MicroART), Department of Veterinary Sciences, University of Trás-os-Montes and Alto Douro (UTAD), 5001-801 Vila Real, Portugal; vanessasilva@utad.pt (V.S.); sara-1603@hotmail.com (S.H.); isabelcarvalho93@hotmail.com (I.C.); 2 Department of Genetics and Biotechnology, University of Trás-os-Montes and Alto Douro (UTAD), 5001-801 Vila Real, Portugal; gigrejas@utad.pt; 3 Functional Genomics and Proteomics Unit, University of Trás-os-Montes and Alto Douro (UTAD), 5001-801 Vila Real, Portugal; 4 Associate Laboratory for Green Chemistry-LAQV, Chemistry Department, Faculty of Science and Technology, University Nova of Lisbon, 2829-516 Lisbon, Portugal; 5 CBQF—Centro de Biotecnologia e Química Fina-Laboratório Associado, Universidade Católica Portuguesa, Escola Superior de Biotecnologia, 4169-005 Porto, Portugal; aferreira@porto.ucp.pt (C.F.); cmanaia@porto.ucp.pt (C.M.M.); 6 Department of Food Hygiene and Technology, Veterinary Faculty, University of León, 24071 León, Spain; rosa.capita@unileon.es (R.C.); carlos.alonso.calleja@unileon.es (C.A.-C.); 7 Institute of Food Science and Technology, University of León, 24071 León, Spain; 8 Veterinary and Animal Science Research Center (CECAV), University of Trás-os-Montes and Alto Douro (UTAD), 5001-801 Vila Real, Portugal; jeduardo@utad.pt (J.E.P.); lmaltez@utad.pt (L.M.); 9 BIOSCOPE Group, LAQV@REQUIMTE, Chemistry Department, Faculty of Science and Technology, NOVA University of Lisbon, 1349-008 Almada, Portugal; jlcm@fct.unl.pt; 10 Proteomass Scientific Society, 2825-466 Costa de Caparica, Portugal

**Keywords:** MRSA, EMRSA-15, MLS_B_, bacteremia, bloodstream infections

## Abstract

In this study we aimed to characterize antimicrobial resistance in methicillin-resistant *Staphylococcus aureus* (MRSA) isolated from bloodstream infections as well as the associated genetic lineages of the isolates. Sixteen MRSA isolates were recovered from bacteremia samples from inpatients between 2016 and 2019. The antimicrobial susceptibility of these isolates was tested by the Kirby–Bauer disk diffusion method against 14 antimicrobial agents. To determine the macrolide–lincosamide–streptogramin B (MLS_B_) resistance phenotype of the isolates, erythromycin-resistant isolates were assessed by double-disk diffusion (D-test). The resistance and virulence genes were screened by polymerase chain reaction (PCR). All isolates were characterized by multilocus sequence typing (MLST), *spa* typing, staphylococcal chromosomal cassette *mec* (SCC*mec*) typing, and accessory gene regulator (*agr*) typing. Isolates showed resistance to cefoxitin, penicillin, ciprofloxacin, erythromycin, fusidic acid, clindamycin, and aminoglycosides, confirmed by the presence of the *bla*Z, *erm*A, *erm*C, *mph*C, *msr*A/B, *aac*(6’)-Ie-*aph*(2’’)-Ia, and *ant*(4’)-Ia genes. Three isolates were Panton–Valentine-leukocidin-positive. Most strains (*n* = 12) presented an inducible MLS_B_ phenotype. The isolates were ascribed to eight *spa*-types (t747, t002, t020, t1084, t008, t10682, t18526, and t1370) and four MLSTs (ST22, ST5, ST105, and ST8). Overall, most (*n* = 12) MRSA isolates had a multidrug-resistance profile with inducible MLS_B_ phenotypes and belonged to epidemic MRSA clones.

## 1. Introduction

*Staphylococcus aureus* is an opportunist human pathogen responsible for numerous types of infections, from skin infections, such as abscesses or infected wounds, to life-threatening conditions, such as endocarditis, osteomyelitis, or septicemia [1]. Some *S. aureus* strains can be quite virulent due to the combined action of several virulence factors, the most important being Panton–Valentine leukocidin (PVL) and toxic shock syndrome toxin, associated with immune evasion, tissue adhesion, and host cell injury [2]. *S. aureus* is known for its ability to acquire antibiotic resistance determinants. In fact, *S. aureus* has become an important cause of nosocomial infections, particularly methicillin-resistant *S. aureus* (MRSA), which is usually associated with a multidrug-resistance profile [3]. Consequently, MRSA infections are difficult to treat and are a leading cause of morbidity and mortality, especially among hospitalized patients and humans with weakened immune systems [4]. Due to the increase of MRSA strains, macrolides, lincosamides, and streptogramin B (MLS_B_) were often used to treat MRSA infections, which led to a subsequent cross-resistance to these antibiotics [5]. Different mechanisms are responsible for the MLS_B_ resistance, the most common being the target modification mediated by the *erm* (erythromycin ribosome methylase) gene [6]. In staphylococci, *erm*A and *erm*C are the main genes conferring the MLS_B_ resistance phenotype, which can be constitutive or inducible [7]. Healthcare-associated MRSA rates vary considerably across countries in Europe, with a high prevalence in Southwest Europe and a lower prevalence in Northeast Europe [8]. The prevalence of MRSA in Portugal has remained one of the highest among the European countries in recent years—around 40% of *S. aureus* isolates from hospitalized individuals with infection in Portugal have been identified as MRSA. The predominant clonal complexes responsible for hospital infections in Portugal are CC22 and CC5, with the epidemic methicillin-resistant *Staphylococcus aureus* 15 (EMRSA-15) clone being the most prevalent [9].

*S. aureus* is considered one of the most important and common pathogens causing bloodstream infections and is the second leading cause of sepsis in industrialized countries [10]. Both hospital- and community-acquired MRSA bacteremia are associated with various clinical manifestations, such as metastatic infections, endocarditis, septic arthritis, osteomyelitis, and septic shock [11]. Community-acquired MRSA bacteremia has now surpassed hospital-acquired bacteremia worldwide, and it is frequently associated with other diseases, such as diabetes, ulcers, or chronic renal disease [12]. Despite the existence of an adequate treatment, MRSA is responsible for mortality rates of 20% to 40% in a period of 30 days [13]. Given the extreme severity of clinical complications from a generalized infection caused by *S. aureus* and its association with resistance to methicillin and most β-lactam antibiotics, it is extremely important to study the genetic characteristics of the most prevalent strains responsible for bacteremia in order to more effectively target the strategies for controlling these infections [14]. This study aimed to isolate and characterize the antimicrobial resistance and genetic lineages of MRSA strains isolated from bloodstream infections.

## 2. Results

A total of 16 MRSA isolates were obtained from 103 hospitalized patients with bacteremia over the 3-year study period, corresponding to a patient incidence of 15.5%. Table 1 shows the genotypical characterization of the MRSA strains. All isolates were resistant to cefoxitin and harbored the *mec*A gene. Eleven isolates belonged to SCC*mec* type IV and five to type II. The isolates were ascribed to eight *spa* types (t747, t002, t020, t1084, t008, t10682, t18526, and t1370). The 16 isolates were grouped into five different sequence types (STs), namely ST22 (*n* = 9), ST5 (*n* = 2), ST105 (*n* = 2), ST8 (*n* = 2), and ST5984 (*n* = 1). ST5984 was first described in this study and differs from ST105 by a one-point mutation on the *arcC* locus. The isolate categorized as ST5984 belonged to *spa*-type t1084, SCC*mec*, and *agr* type II and presented resistance to penicillin, erythromycin, ciprofloxacin, and fusidic acid.

The majority of the isolates (*n* = 9) were typed as ST22 and SCC*mec* IV, also known as the EMRSA-15 clone. Six of these isolates were *spa*-type t747, and the other three were t020, t18526, and t1370. EMRSA-15 isolates were resistant to penicillin, and eight out of nine harbored the *bla*Z gene. Seven isolates showed resistance to erythromycin, and three were coresistant to clindamycin, showing a constitutive MLS_B_ (cMLS_B_) phenotype. Four erythromycin-resistant isolates did not show clindamycin resistance; however, they were positive upon D-testing and were considered inducible MLS_B_ (iMLS_B_) isolates. The observed MLS_B_ phenotypes were mostly determined by identification of combinations of two or more genes: *erm*C + *msr*(A/B) (*n* = 1), *erm*A + *mph*C (*n* = 1), *erm*A + *erm*C + *msr*(A/B) (*n* = 1), and *erm*C + *msr*(A/B) + *mph*C (*n* = 4). Only one isolate was resistant to aminoglycosides, namely gentamicin and tobramycin, and harbored the resistance genes *aac*(6’)-Ie-*aph*(2’’)-Ia and *ant*(4’)-Ia. Regarding the virulence factors, three isolates were PVL-positive, all isolates harbored the genes encoding hemolysins, and eight isolates carried the *eta* gene. All EMRSA-15 isolates belonged to *agr* type I.

Two isolates were typed as ST5-SCC*mec* II and one isolate as ST105-SCC*mec* II (New York/Japan and New York/Japan (related) clones, respectively). Both isolates were ascribed to *spa*-type t002. These isolates showed resistance to penicillin and ciprofloxacin and harbored the *bla*Z resistance gene. Two isolates showed an iMLS_B_ phenotype and carried the gene combinations of *erm*A + *msr*(A/B) and *msr*(A/B) + *mph*C. One isolate carried the *aac*(6’)-Ie-*aph*(2’’)-Ia gene encoding resistance to gentamicin. None of the isolates were positive for PVL; nevertheless, all isolates were positive for genes encoding hemolysins, and all were *agr* type II.

Finally, two isolates belonged to ST8 and SCC*mec* type IV (variant of the USA300 clone). Both isolates were typed as t008. One of the isolates showed a multidrug-resistant phenotype with resistance to penicillin, ciprofloxacin, and erythromycin and inducible resistance to clindamycin, harboring the respective resistance genes *bla*Z, *erm*C, *msr*(A/B), and *mph*C. The second isolate showed resistance to penicillin and fusidic acid. Both isolates belonged to *agr* type I and carried the genes encoding alpha- and beta-hemolysins.

## 3. Discussion

A total of 103 cases of bacteremia were identified at the local hospital between 2016 and 2019, of which 15.5% were caused by MRSA. All isolates were typically epidemic hospital-acquired MRSA (HA-MRSA) clones. MRSA bacteremia has been reported worldwide, and its frequency varies from one country to another. In 2018, the percentage of invasive MRSA in bacteremia in different parts of Europe varied from 0.0% to 43.0% with an average of 16.4% [15]. Notably, in Southern European countries, such as Italy, Romania, Greece, and Cyprus, these rates were higher than in other European countries. In Portugal, the percentage of invasive MRSA in bacteremia was 38.1%. MRSA bacteremia treatment is challenging, especially when dealing with multidrug-resistant strains. Indeed, in our study, 12 of the 16 isolates were considered multidrug-resistant since they presented resistance to antibiotics belonging to at least three distinct classes of antimicrobials. 

EMRSA-15 is one of the most recurrent HA-MRSA clonal lineages in recent years [16,17]. This clone is known for its rapid spread and is responsible for causing several invasive infections, such as bacteremia [18]. EMRSA-15 carries SCC*mec* type IV, which is frequent in HA-MRSA clones and is smaller and has a lower fitness cost compared to SCC*mec* types II and III, increasing the clone’s ability to spread worldwide [19]. In 2013, a study conducted by Faria et al. reported that EMRSA-15, followed by ST105-II, was the dominant clone among MRSA bloodstream infections in Portugal [20]. EMRSA-15 was also the predominant clone found in our study. Since 2001, this clone has been repeatedly isolated in hospitalized patients, communities, the environment, and animals in Portugal, replacing the resident HA-MRSA clones and becoming the main clone in this country [21]. Initially, the EMRSA-15 clone in Portugal was characterized by *spa*-types t747, t032, and t2357; however, as EMRSA-15 became the main clone, there was an increase in *spa* diversity [20]. Nevertheless, in this study, *spa*-type t747 was the most common. One of the EMRSA-15 isolates belonged to *spa*-type t020, which is a well-established type in Germany and is highly associated with EMRSA-15 [22,23]. *spa*-types t18526 and t1370 were also detected in this study, each in one isolate. The *spa*-type t18526 was reported for the first time in one of our previous studies conducted with samples from the same hospital with MRSA strains isolated from infected diabetic foot ulcers, in which the most prevalent clone and *spa*-type were also EMRSA-15 and t747 [9]. As for t1370, it was the predominant clone in an outbreak in a neonatal unit in the UK and in human patients in New Zealand and was always associated with EMRSA-15 [24,25]. The presence of PVL-encoding genes was only detected in EMRSA-15 isolates. Similar results were obtained by Goudarzi et al. when studying the molecular characteristics of MRSA strains from patients with bacteremia [26]. Although the PVL toxin is often associated with skin and soft-tissue infections, studies have shown an association between PVL and severe invasive infections [27]. PVL and SCC*mec* type IV presence are often used as markers of community-associated MRSA (CA-MRSA). This assumption may be inaccurate since it frequently includes the EMRSA-15 and USA800 clones, which are epidemiologically HA-MRSA [18]. The *eta* gene was found only among EMRSA-15 isolates, and consistent with other studies, all *eta* genes belonged to SCC*mec* type IV [26]. Furthermore, the *etb* gene was not detected in our study, which is in accordance with other studies that showed that invasive MRSA strains carried the *eta* gene but few or none carried the *etb* gene [26,28]. All EMRSA-15 isolates in our study belonged to *agr* type I, in agreement with other studies in which *agr* I, followed by *agr* II, was the most common type in MRSA bacteremia [29,30]. Furthermore, Ben Ayed et al. showed that *agr* I was associated with invasive infections, bacteremia in particular [31]. However, *agr* locus is strongly associated with bacterial genetic background, and therefore its prevalence may be driven by the genotypes circulating in each hospital. Goudarzi et al. reported that MRSA bacteremia isolates belonging to SCC*mec* IV and II isolates were distributed among *agr* type III [26]. In another study, *agr* II was the most common *agr* type in MRSA bacteremia strains, followed by *agr* I; however, the majority of strains belonged to CC5, which suggests that *agr* type may also be associated with clonal complex [32]. Indeed, Aschbacher et al. showed that bacteremia isolates belonging to CC22, CC5, and CC8 were *agr* types I, II, and I, respectively [30]. These results are in accordance with our study, in which all USA300 isolates belonged to *agr* type I, and all New York/Japan clones were *agr* type II. Three New York/Japan (or related) clones were detected in bacteremia isolates. This clone has been reported to be associated with bacteremia and is the most prevalent in France and South Korea [33,34]. The New York/Japan clone and ST105-MRSA-II were also found in bacteremia isolates in the study by Faria et al., who suggested that the ST105-MRSA-II clone could replace EMRSA-15 and be the next clonal wave of MRSA in Portuguese hospitals [20]. Nevertheless, this shift was not confirmed, since our studies and other recent studies in Portugal showed that there was no modification in the predominance of EMRSA-15 [9]. Furthermore, the New York/Japan clone seems to be significantly decreasing in prevalence in hospitals [35,36]. *spa*-type t002 is strongly linked with ST105-MRSA-II, since most New York/Japan isolates reported are type t002. Two ST8-MRSA-IV clones were also isolated in our study. These clones are a variant of the epidemic clone USA300, since this clone is linked to carriage of PVL and both of our isolates were PVL-negative. USA300 is sporadically isolated in MRSA infections in Portugal; this clone is frequently found in the United States (*spa-*type t008), where it is often responsible for bacteremia, and is found to a much lesser extent in Europe and the rest of the world [37].

Twelve of the sixteen isolates were resistant to erythromycin; however, only three showed coresistance to clindamycin and as such were categorized as cMLS_B_. Those nine isolates were further characterized by D-test to the MLS_B_ phenotype. All showed the inducible MLS_B_ phenotype. Both cMLS_B_ and iMLS_B_ harbored several combinations of genes conferring resistance to macrolides and lincosamides; however, *erm*A, *erm*C, or both were present in all isolates. The *erm*A and *erm*C genes are the genes most commonly found in MLS_B_-resistant *S. aureus*; nevertheless, MLS_B_-resistant MRSA often carries combinations of two or more resistance genes [7,38]. In staphylococci showing an iMLS_B_ phenotype, the methylase mRNA produced by bacteria is inactive, and the activation only occurs in the presence of a macrolide. In contrast, in the cMLS_B_-resistance phenotype, active methylase mRNA is produced in the absence of a macrolide [38]. Therefore, identifying the MLS_B_-resistance phenotype is essential since strains presenting an iMLS_B_ phenotype may switch to a cMLS_B_ phenotype under antibiotic pressure, which may lead to treatment failure [38].

## 4. Material and Methods

### 4.1. Bacterial Isolates

Blood samples were collected from 103 inpatients with bacteremia infection hospitalized at the Hospital Centre of Trás-os-Montes e Alto Douro E.P.E., Vila Real, Portugal, from 2016 to 2019. A small volume of blood culture was inoculated on an oxacillin-resistance-screening agar base (ORSAB) (OXOID) supplemented with 2 mg/L of oxacillin to isolate MRSA strains and incubated at 37 °C for 24 h. Four colonies from each plate were recovered and seeded onto Baird–Parker agar plates for further identification of possible *S. aureus*. MRSA strains were identified based on Gram staining, biochemical tests (catalase, DNase, and coagulase), and genotyping.

### 4.2. Antimicrobial Resistance Profile

MRSA strains were characterized according to their antibiotic resistance profiles using the Kirby–Bauer disk-diffusion method against 14 antimicrobial agents: cefoxitin (30 µg), chloramphenicol (30 µg), ciprofloxacin (5 µg), clindamycin (2 µg), erythromycin (15 µg), fusidic acid (10 µg), gentamicin (10 µg), kanamycin (30 µg), linezolid (10 µg), mupirocin (200 µg), penicillin (1U), tetracycline (30 µg), tobramycin (10 µg), and trimethoprim/sulfamethoxazole (1.25/23.75 µg). The tests were performed according to the European Committee on Antimicrobial Susceptibility Testing (EUCAST, 2018) guidelines except the test for kanamycin, which followed Clinical and Laboratory Standards Institute standards (CLSI, 2017). Isolates showing resistance to erythromycin were further characterized by double-disk diffusion (D-test) to determine the MLS_B_ phenotype. Briefly, erythromycin and clindamycin disks were placed onto inoculated Muller–Hinton plates 15 mm apart from edge to edge. If the inhibition zone around the clindamycin disk showed a D-shape, the isolate was considered to have an inducible MLS_B_ (iMLS_B_) phenotype. Resistance to both erythromycin and clindamycin indicated a constitutive MLS_B_ (cMLS_B_) phenotype [39]. Quality control was performed with *S. aureus* strain ATCC 25923.

DNA was extracted from fresh cultures as previously described [9]. Briefly, two colonies of fresh cultures from each isolate were suspended in 45 µL of Milli-Q water. Five microliters of lysostaphin (1 mg/mL) was added, and the samples were incubated for 10 min at 37 °C. Then, 150 µL of Tris-HCl (0.1 M), 45 μL of Milli-Q water, and 5 μL of proteinase K (2 mg/mL) were added, and the samples were incubated at 67 °C for 10 min. Lastly, the samples were boiled for 5 min at 100 °C.

According to the phenotypic resistance of each isolate, the presence of the following antibiotic resistance genes was investigated by polymerase chain reaction (PCR): *bla*Z, *erm*(A), *erm*(B), *erm*(C), *erm*(T), *erm*(Y), *msr*(A/B), *mph*C, *lin*B, *vga*B, *vga*C, *aac*(6’)-Ie-*aph*(2’’)-Ia, *ant*(4’)-Ia, *fus*B, and *fus*C (Supplementary Table S1).

### 4.3. Characterization of Virulence Factors

The presence of the virulence genes encoding alpha- and beta-hemolysins (hla and hlb), exfoliative toxins (eta and etb), toxic shock syndrome toxin (tst), and Panton–Valentine leucocidin (PVL) (*lukF/lukS*-PV) was determined by PCR (Supplementary Table S1). Positive and negative controls used in all experiments belonged to the strain collection of University of Trás-os-Montes and Alto Douro.

### 4.4. Molecular Characterization

Multilocus sequence typing (MLST) and *spa* typing were performed for all isolates as previously described, supported by the public databases MLST and the Ridom SpaServer. According to the sequence type (ST), each isolate was grouped according to the corresponding clonal complex (CC). All isolates were characterized by *agr* typing and staphylococcal chromosomal cassette *mec* (SCC*mec*) typing (I–V) using specific primers (Supplementary Table S1).

## 5. Conclusions

We found epidemic HA-MRSA clones, namely EMRSA-15, USA300, and New York/Japan, in samples recovered from bloodstream infections over a period of 3 years. Our results corroborate the relatively high prevalence of EMRSA-15 circulating in Portuguese hospitals. Most isolates were multidrug-resistant and presented an iMLSB or cMLSB phenotype, which may result in a therapeutic problem of inadequacy of antibiotic treatment and lead to high morbidity and mortality.

## Figures and Tables

**Table 1:** Antimicrobial resistance, virulence factors, and molecular characteristics of methicillin-resistant *Staphylococcus aureus* (MRSA) strains isolated from blood cultures.

Isolate	Antimicrobial Resistance		Virulence	Molecular Typing			
	Phenotype	Genotype ^a^		MLST (CC)	*spa*	SCC*mec*	*agr*
**VS2761**	FOX, PEN, ERY, DA ^1^, CIP	*mec*A, *erm*C, *msr*(A/B)	*hl*A	22 (22)	t747	IV	I
**VS2762**	FOX, PEN, ERY, DA ^2^, CN, CIP	*mec*A, *bla*Z, *erm*A, *msr*(A/B), *aac*(6’)-Ie-*aph*(2’’)-Ia	*hl*A	105 (5)	t002	II	II
**VS2763**	FOX, PEN, CIP	*mec*A, *bla*Z	*hlA*, *hl*B, *etA*	22 (22)	t747	IV	I
**VS2764**	FOX, PEN, ERY, DA ^2^, CIP	*mec*A, *bla*Z, *erm*C, *msr*(A/B), *mph*C	*hl*A, *etA*	22 (22)	t747	IV	I
**VS2765**	FOX, PEN, ERY, DA ^2^, CIP	*mec*A, *bla*Z, *erm*C, *msr*(A/B), *mph*C	*lukF/lukS-*PV, *hl*A, *hl*B, *et*A	22 (22)	t747	IV	I
**VS2766**	FOX, PEN, ERY, DA ^1^, CN, TOB, CIP	*mec*A, *bla*Z, *erm*C, *msr*(A/B), *mph*C, *aac*(6’)-Ie-*aph*(2’’)-Ia, *ant*(4’)-Ia	*lukF/lukS-*PV, *hl*A, *hl*B, *etA*	22 (22)	t020	IV	I
**VS2767**	FOX, PEN, CIP	*mec*A, *bla*Z	*hl*A, *hl*B, *etA*	22 (22)	t747	IV	I
**VS2768**	FOX, PEN, ERY, DA ^2^, CIP	*mec*A, *bla*Z, *erm*C, *msr*(A/B), *mph*C	*lukF/lukS-*PV, *hl*A, *etA*	22 (22)	t747	IV	I
**VS2769**	FOX, PEN, ERY, DA ^2^, CIP	*mec*A, *bla*Z, *msr*(A/B), *mph*C	*hl*A, *hl*B	5 (5)	t002	II	II
**VS2770**	FOX, PEN, ERY, DA ^2^, CIP, FD	*mec*A, *bla*Z, *erm*A, *erm*C, *msr*(A/B), *mph*C	*hl*A, *hl*B	5984	t1084	II	II
**VS2771**	FOX, PEN, ERY, DA ^2^, CIP	*mec*A, *bla*Z, *erm*C, *msr*(A/B), *mph*C	*hl*A	8 (8)	t008	IV	I
**VS2772**	FOX, PEN, CIP	*mec*A, *bla*Z	*hl*B	5 (5)	t002	II	II
**VS2773**	FOX, PEN, ERY, DA ^2^, CIP	*mec*A, *bla*Z, *erm*A, *erm*C, *msr*(A/B), *mph*C	*hl*B	105 (5)	t10682	II	II
**VS2774**	FOX, PEN, ERY, DA ^1^, CIP	*mec*A, *bla*Z, *erm*A, *mph*C	*hl*B, *etA*	22 (22)	t18526	IV	I
**VS2775**	FOX, PEN, ERY, DA ^2^, CIP	*mec*A, *bla*Z, *erm*A, *erm*C, *msr*(A/B)	*hl*B, *etA*	22 (22)	t1370	IV	I
**VS2776**	FOX, PEN, FD	*mec*A, *bla*Z	*hl*B	8 (8)	t008	IV	I

^1^ Constitutive MLS_B_ (cMLS_B_) phenotype; ^2^ Inducible MLS_B_ (iMLS_B_) phenotype; FOX: cefoxitin; PEN: penicillin; ERY: erythromycin; DA: clindamycin; CN: gentamicin; TOB: tobramycin; CIP: ciprofloxacin; FD: fusidic acid; MLST: multilocus sequence typing; ST: sequence type; CC: clonal complex; SCC*mec*: staphylococcal cassette chromosome *mec;* ^a^ *mec*A gene encodes the protein PBP2A; *bla*Z encodes the protein BlaZ; *erm* genes encode the rRNA adenine N-6-methyltransferase, *msr*(A/B) encodes the peptide methionine sulfoxide reductase; *mph*C encodes the macrolide 2’-phosphotransferase; *aac*(6’)-Ie-*aph*(2’’)-Ia encodes the bifunctional enzyme AAC/APH; and *ant*(4’)-Ia encodes the aminoglycoside O-nucleotidyltransferase ANT(4’)-Ia

## Supplementary Materials

The following are available online at https://www.mdpi.com/2079-6382/9/7/375/s1: Table S1: Primers used for molecular typing and detection of antimicrobial resistance genes in MRSA strains.
