# Supplementary material for: Genetic Characterization of Methicillin-Resistant Staphylococcus aureus Isolates from Human Bloodstream Infections: Detection of MLSB Resistance
Source: Antibiotics (Basel). 2020 Jul 3;9(7):375. doi: 10.3390/antibiotics9070375 (PMC7400445; doi:10.3390/antibiotics9070375)
Supplement: Supplementary file 1 [file antibiotics-09-00375-s001.pdf]

Table 1. Primers used for molecular typing and detection of antimicrobial resistance genes in MRSA strains.

Target gene	Primer sequence (5'-3')	Amplicon size (bp)	Reference
	**Antimicrobial resistance**		
mecA	F: GGGATCATAGCGTCATTATTC<br>R: AACGATTGTGACACGATAGCC	527	[1]
nuc	F: TCAGCAAATGCATCACAAACAG<br>R: CGTAAATGCACTTGCTTCAGG	255	[1]
**rDNA 16S**	F: GTGCCAGCAGCCGCGGTAA<br>R: AGACCCGGGAACGTATTCAC	886	[1]
blaZ	F: CAGTTCACATGCCAAAGAG<br>R: TACACTCTTGGCGGTTTC	772	[2]
***erm*(A)**	F: TCTAAAAAGCATGTAAAAGAA<br>R: CTTCGATAGTTTATTAATATTAG	645	[3]
***erm*(B)**	F: GAAAAGTACTCAACCAAATA<br>R: AGTAACGGTACTTAAATTGTTTA	639	[3]
***erm*(C)**	F: TCAAAACATAATATAGATAAA<br>R: GCTAATATTGTTTAAATCGTCAAT	642	[3]
***erm*(T)**	F: CCGCCATTGAAATAGATCCT<br>R: TTCTGTAGCTGTGCTTTCAAAAA	200	[4]
***erm*(Y)**	F: AGGCCCCTTTTAAAGACGAAGGCA<br>R: GGCGCGATTGTTCATTTTAAGGCCC	320	[4]
***msr*(A/B)**	F: GCAAATGGTGTAGGTAAGACAACT<br>R: ATCATGTGATGTAAACAAAAT	399	[5]
***mph*(C)**	F: ATGACTCGACATAATGAAAT<br>R: CTACTCTTTCATACCTAACTC	900	[2]
***lin*(B)**	F: CCTACCTATTGTTTGTGGAA<br>R: ATAACGTTACTCTCCTATTC	944	[6]
***vga*(B)**	F: TGACAATATGAGTGGTGGTG<br>R: GCGACCATGAAATTGCTCTC	579	[7]
***vga*(C)**	F: ACGAGGGGACAATCACGCCG<br>R: TTGGTCGTCGGTTCGTCTGC<br>R: CAATATCACCAGAGCAGGCT	159	[4]
aac(6′)-Ie-aph(2′′)-Ia	F: CCAAGAGCAATAAGGGCATA<br>R: CACTATCATAACCACTACCG	220	[8]
aph(3′)-IIIa	F: GCCGATGTGGATTGCGAAAA<br>R: GCTTGATCCCCAGTAAGTCA	292	[8]
ant(4′)-Ia	F: GCAAGGACCGACAACATTTC<br>R: TGGCACAGATGGTCATAACC	165	[8]
***fus*B**	F: CTATAATGATATTAATGAGATTTTTGG<br>R: TTTTTACATATTGACCATCCGAATTGG	431	[9]
***fus*C**	F: TTAAAGAAAAAGATATTGATATCTCGG<br>R: TTTACAGAATCCTTTTACTTTATTTGG	332	[9]
	**Virulence**		
hla	F: CTGATTACTATCCAAGAAATTCGATTG<br>R: CTTTCCAGCCTACTTTTTTATCAGT	209	[10]
hlb	F: GTGCACTTACTGACAATAGTGC	309	[10]

	R: GTTGATGAGTAGCTACCTTCAGT		
eta	F: ACTGTAGGAGCTAGTGCATTTGT<br>R: TGGATACTTTTGTCTATCTTTTTCATCAAC	190	[10]
etb	F: CAGATAAAGAGCTTTATACACACATTAC<br>R: AGTGAACTTATCTTTCTATTGAAAAACACTC	612	[10]
tst	F: TTCACTATTTGTAAAAGTGTCAGACCCACT<br>R: TACTAATGAATTTTTTTATCGTAAGCCCTT	180	[10]
***lukF*/*lukS*-PV**	F: ATCATTAGGTAAAATGTCTGGACATGATCCA<br>R: GCATCAAGTGTATTGGATAGCAAAAGC	443	[11]
	**Molecular typing**		
spa	F: AGACGATCCTTCGGTGAGC<br>R: GCTTTTGCAATGTCATTTACTG	Variable	[12]
arcC	F: TTGATTCACCAGCGCGTATTGTC<br>R: AGGTATCTGCTTCAATCAGCG	456	[13]
aroE	F: ATCGGAAATCCTATTTCACATTC<br>R: GGTGTTGTATTAATAACGATATC	456	[13]
glpF	F: CTAGGAACTGCAATCTTAATCC<br>R: TGGTAAAATCGCATGTCCAATTC	465	[13]
gmk	F: ATCGTTTTATCGGGACCATC<br>R: TCATTAACTACAACGTAATCGTA	429	[13]
pta	F: GTTAAAATCGTATTACCTGAAGG<br>R: GACCCTTTTGTTGAAAAGCTTAA	474	[13]
tpi	F: TCGTTCATTCTGAACGTCGTGAA<br>R: TTTGCACCTTCTAACAATTGTAC	402	[13]
yquiL	F: CAGCATACAGGACACCTATTGGC<br>R: CGTTGAGGAATCGATACTGGAAC	516	[13]
***agr*I**	F: GTCACAAGTACTATAAGCTGCGAT<br>R: ATGCACATGGTGCACATGC	440	[14]
***agr*II**	F: GTATTACTAATTGAAAAGTGCCATAGC<br>R: ATGCACATGGTGCACATGC	572	[14]
***agr*III**	F: CTGTTGAAAAAGTCAACTAAAAGCTC<br>R: ATGCACATGGTGCACATGC	406	[14]
***agr*IV**	F: CGATAATGCCGTAATACCCG<br>R: ATGCACATGGTGCACATGC	656	[14]
**SCC*mec* I**	F: GCTTTAAAGAGTGTCGTTACAGG<br>R: GTTCTCTCATAGTATGACGTCC	613	[15]
**SCC*mec* II**	F: CGTTGAAGATGATGAAGCG<br>R: CGAAATCAATGGTTAATGGACC	398	[15]
**SCC*mec* III**	F: CCATATTGTGTACGATGCG<br>R: CCTTAGTTGTCGTAACAGATCG	280	[15]
**SCC*mec* IVa**	F: GCCTTATTCGAAGAAACCG<br>R: CTACTCTTCTGAAAAGCGTCG	776	[15]
**SCC*mec* IVb**	F: TCTGGAATTACTTCAGCTGC<br>R: AAACAATATTGCTCTCCCTC	493	[15]
**SCC*mec* IVc**	F: ACAATATTTGTATTATCGGAGAGC<br>R: TTGGTATGAGGTATTGCTGG	200	[15]
**SCCmec IVd**	F: CTCAAAATACGGACCCCAATACA<br>R: TGCTCCAGTAATTGCTAAAG	881	[15]

**SCCmec V**	F: GAACATTGTTACTTAAATGAGCG<br>R: TGAAAGTTGTACCCTTGACACC	325	[15]

References

1. Zhang, K.; Sparling, J.; Chow, B.L.; Elsayed, S.; Hussain, Z.; Church, D.L.; Gregson, D.B.; Louie, T.; Conly, J.M. New quadriplex PCR assay for detection of methicillin and mupirocin resistance and simultaneous discrimination of Staphylococcus aureus from coagulase-negative staphylococci. *J. Clin. Microbiol.* **2004**, *42*, 4947–4955.
2. Schnellmann, C.; Gerber, V.; Rossano, A.; Jaquier, V.; Panchaud, Y.; Doherr, M.G.; Thomann, A.; Straub, R.; Perreten, V. Presence of New mecA and mph(C) Variants Conferring Antibiotic Resistance in Staphylococcus spp. Isolated from the Skin of Horses before and after Clinic Admission. *J. Clin. Microbiol.* **2006**, *44*, 4444–4454.
3. Sutcliffe, J.; Grebe, T.; Tait-Kamradt, A.; Wondrack, L. Detection of Rythromycin-Resistant Determinants by PCR. *Antimicrob. Agents Chemother.* **1996**, *40*, 2562–2566.
4. Gómez-Sanz, E.; Torres, C.; Lozano, C.; Fernández-Pérez, R.; Aspiroz, C.; Ruiz-Larrea, F.; Zarazaga, M. Detection, Molecular Characterization, and Clonal Diversity of Methicillin-Resistant Staphylococcus aureus CC398 and CC97 in Spanish Slaughter Pigs of Different Age Groups. *Foodborne Pathog. Dis.* **2010**, *7*, 1269–1277.
5. Wondrack, L.; Massa, M.; Yang, B. V.; Sutcliffe, J. Clinical strain of Staphylococcus aureus inactivates and causes efflux of macrolides. *Antimicrob. Agents Chemother.* **1996**, *40*, 992–998.
6. Bozdogan, B.; Berrezouga, L.; Kuo, M.S.; Yurek, D.A.; Farley, K.A.; Stockman, B.J.; Leclercq, R. A new resistance gene, linB, conferring resistance to lincosamides by nucleotidylation in Enterococcus faecium HM1025. *Antimicrob. Agents Chemother.* **1999**, *43*, 925–929.
7. Hammerum, A.M.; Jensen, L.B.; Aarestrup, F.M. Detection of the satA gene and transferability of virginiamycin resistance in Enterococcus faecium from food-animals. *FEMS Microbiol. Lett.* **1998**, *168*, 145–151.
8. Van de Klundert, J.A.M.; Vliegenthart, J.. PCR detection of genes coding for aminoglycoside-modifying enzymes. In *Diagnostic Molecular Microbiology: Principles and Applications*; Persing, D.H., Smith, T.F., Tenover, F.C., White, T.J., Eds.; American Society for Microbiology: Washington DC, 1993.
9. McLaws, F.; Chopra, I.; O'Neill, A.J. High prevalence of resistance to fusidic acid in clinical isolates of Staphylococcus epidermidis. *J. Antimicrob. Chemother.* **2008**, *61*, 1040–1043.
10. Jarraud, S.; Mougel, C.; Thioulouse, J.; Lina, G.; Meugnier, H.; Forey, F.; Etienne, J.; Vandenesch, F.; Nesme, X. Relationships between Staphylococcus aureus Genetic Background, Virulence Factors, agr Groups (Alleles), and Human Disease. *Infect. Immun.* **2002**, *70*, 631–641.
11. Lina, G.; Piemont, Y.; Godail-Gamot, F.; Bes, M.; Peter, M.-O.; Gauduchon, V.; Vandenesch, F.; Etienne, J. Involvement of Panton-Valentine Leukocidin - Producing Staphylococcus aureus in Primary Skin Infections and Pneumonia. *Clin. Infect. Dis.* **1999**, *29*, 1128–1132.
12. Shopsin, B.; Gomez, M.; Montgomery, S.O.; Smith, D.H.; Waddington, M.; Dodge, D.E.; Bost, D.A.; Riehman, M.; Naidich, S.; Kreiswirth, B.N. Evaluation of protein A gene polymorphic region DNA sequencing for typing of Staphylococcus aureus strains. *J. Clin. Microbiol.* **1999**, *37*, 3556–3563.
13. Enright, M.C.; Day, N.P.; Davies, C.E.; Peacock, S.J.; Spratt, B.G. Multilocus sequence typing for characterization of methicillin-resistant and methicillin-susceptible clones of Staphylococcus aureus. *J. Clin. Microbiol.* **2000**, *38*, 1008–1015.
14. Shopsin, B.; Mathema, B.; Alcabes, P.; Said-Salim, B.; Lina, G.; Matsuka, A.; Martinez, J.; Kreiswirth, B.N. Prevalence of agr Specificity Groups among Staphylococcus aureus Strains Colonizing Children and Their Guardians. *J. Clin. Microbiol.* **2003**, *41*, 456 LP–459.
15. Zhang, K.; McClure, J.-A.; Elsayed, S.; Louie, T.; Conly, J.M. Novel Multiplex PCR Assay for Characterization and Concomitant Subtyping of Staphylococcal Cassette Chromosome mec Types I to V in Methicillin-Resistant Staphylococcus aureus *J. Clin. Microbiol.* **2005**, *43*, 5026 LP–5033.
